# A Comparison of Microfluidic-Jet Spray Drying, Two-Fluid Nozzle Spray Drying, and Freeze-Drying for Co-Encapsulating *β*-Carotene, Lutein, Zeaxanthin, and Fish Oil

**DOI:** 10.3390/foods10071522

**Published:** 2021-07-01

**Authors:** Yongchao Zhu, Yaoyao Peng, Jingyuan Wen, Siew Young Quek

**Affiliations:** 1 Food Science, School of Chemical Sciences, The University of Auckland, Auckland 1010, New Zealand; yzhu313@aucklanduni.ac.nz (Y.Z.); ypen083@aucklanduni.ac.nz (Y.P.); 2 School of Pharmacy, Faculty of Medical and Health Sciences, The University of Auckland, Auckland 1023, New Zealand; j.wen@auckland.ac.nz; 3 Riddet Institute, Massey University, Palmerston North 4442, New Zealand

**Keywords:** microfluidic-jet spray drying, co-encapsulation, *β*-carotene, lutein, zeaxanthin, fish oil, in vitro digestion

## Abstract

Various microencapsulation techniques can result in significant differences in the properties of dried microcapsules. Microencapsulation is an effective approach to improve fish oil properties, including oxidisability and unpleasant flavour. In this study, *β*-carotene, lutein, zeaxanthin, and fish oil were co-encapsulated by microfluidic-jet spray drying (MFJSD), two-fluid nozzle spray drying (SD), and freeze-drying (FD), respectively. The aim of the current study is to understand the effect of different drying techniques on microcapsule properties. Whey protein isolate (WPI) and octenylsuccinic anhydride (OSA) modified starch were used as wall matrices in this study for encapsulating carotenoids and fish oil due to their strong emulsifying properties. Results showed the MFJSD microcapsules presented uniform particle size and regular morphological characteristics, while the SD and FD microcapsules presented a large distribution of particle size and irregular morphological characteristics. Compared to the SD and FD microcapsules, the MFJSD microcapsules possessed higher microencapsulation efficiency (94.0–95.1%), higher tapped density (0.373–0.652 g/cm^3^), and higher flowability (the Carr index of 16.0–30.0%). After a 4-week storage, the SD microcapsules showed the lower retention of carotenoids, as well as *ω*-3 LC-PUFAs than the FD and MFJSD microcapsules. After in vitro digestion trial, the differences in the digestion behaviours of the microcapsules mainly resulted from the different wall materials, but independent of drying methods. This study has provided an alternative way of delivering visual-beneficial compounds via a novel drying method, which is fundamentally essential in both areas of microencapsulation application and functional food development.

## 1. Introduction

Visual impairment and blindness greatly reduce the quality of life, and pose huge socioeconomic burdens to families due to the high costs of public health treatments [1]. According to the World Health Organisation (WHO)’s World Report on Vision 2019 [2], at least 2.2 billion people globally have vision impairments or blindness; however, from this number, at least 1 billion people have vision impairments that could have been prevented or have not been addressed. Therefore, seeking alternative ways to prevent or assist with treating visual problems has long been a popular topic.

Some lipophilic bioactives were reported to be effective at preventing common ocular diseases, including cataracts and age-related macular degeneration (AMD). Among them, *β*-carotene has gained much attention for its high provitamin A activity, which can prevent the ocular diseases related to vitamin A deficiency [3,4]. In addition, lutein and zeaxanthin are capable of preventing macular degeneration and cataracts, and have a filtering effect on harmful blue light to prevent photooxidation [5,6]. Fish oil, on the other hand, is a rich source of *ω*-3 long chain polyunsaturated fatty acids (*ω*-3 LC-PUFAs), including eicosapentaenoic acid (EPA, C20:5n-3) and docosahexaenoic acid (DHA, C22:6n-3) [7]. Studies reported that fish oil is effective in the treatment of dry eye disease and AMD [8,9,10]. Nevertheless, the inherent chemical reactivities of these lipophilic compounds make them sensitive to oxygen, light, and heat, which greatly limit their applications in functional foods or pharmaceuticals [11]. Thus, an effective way of delivering these functional compounds is of great interest to global researchers.

Microencapsulation is a feasible approach to preserve the chemical integrity and physiological benefits of bioactive compounds. There are many microencapsulation techniques available and each technique has its own characteristics. The two-fluid nozzle spray drying (SD) and freeze-drying (FD) have been widely applied in the microencapsulation of lipophilic bioactives. The two-fluid nozzle spray dryer produces polydisperse droplets and dehydrates the fine droplets by hot air, while freeze-drying, also known as lyophilisation, is a drying technique to remove water from heat-sensitive materials at low temperature [12,13].

The microfluidic-jet spray drying (MFJSD) is a relatively novel spray-drying technique that produces monodisperse microparticles with narrow size distribution and uniform morphology [14]. MFJSD is equipped with a monodisperse droplet generator, which is capable of atomizing the feed solution into monodispersed droplets with a vertical droplet trajectory [15]. Each individual droplet could be ensured of an identical drying history and, thus, the variable of droplet size can be effectively minimised [16]. The application of MFJSD can reduce the complexity of spray-dried droplets and decrease the possibility of powder agglomeration to improve the flowability of dried particles. At present, the studies on bioactive encapsulation by MFJSD are still quite limited, and none of them has reported on the co-encapsulation of carotenoids and fish oil.

This study aims to understand the drying technique effects on the properties, storage stability, and digestibility of the microcapsules containing three carotenoids (*β*-carotene, lutein, zeaxanthin), and fish oil. This is also the first study on a systematic comparison among the MFJSD, SD, and FD, regarding their feasibility and efficiency in co-encapsulating carotenoids and fish oil.

## 2. Materials and Methods

### 2.1. Samples and Chemicals

Fish oil (Incromega TG3322) was kindly donated by Croda International (Snaith, England, UK). Carotenoids, including *β*-carotene, lutein, and zeaxanthin were provided as gifts by DSM Nutritional Products (Heerlen, Netherlands). Whey protein isolate (WPI) was supplied by Fonterra Co-operative Group Limited (Auckland, New Zealand). Octenylsuccinic anhydride (OSA) modified starch (HI-CAP100) was purchased from Ingredion (Westchester, IL, USA). Methyl heptadecanoate, butylated hydroxytoluene (BHT) was obtained from Sigma-Aldrich (St. Louis, MO, USA). Reagents of electrolyte stock solutions for in vitro digestion were as follows: sodium carbonate, calcium chloride dehydrate, sodium chloride, sodium hydrogen carbonate, potassium phosphate and potassium chloride from ECP Ltd. (Auckland, New Zealand); magnesium chloride hexahydrate from Scharlau (Barcelona, Spain); ammonium carbonate from Ajax FineChem (Wollongong, Australia); α-amylase and pepsin (from porcine gastric mucosa), pancreatin (from porcine pancreas), and bile salts from Sigma-Aldrich (St. Louis, MO, USA). Nile Blue for oil stain was obtained from Sigma-Aldrich (St. Louis, MO, USA). All chemicals used in this study were of analytical grade.

### 2.2. Preparation of Emulsion

WPI and OSA modified starch was dissolved in water with constant stirring at 55 °C and 80 °C, respectively, for 60 min, using a magnetic hot plant (IKA RCT basic, German), followed by stirring overnight at room temperature for complete dispersion. An oil phase containing fish oil (99.55%), *β*-carotene (0.15%), lutein (0.15%), and zeaxanthin (0.15%) was firstly prepared before it was mixed with the above aqueous phase to prepare an emulsion with 20% (*w*/*w*) total solid content (16% *w*/*w* wall materials and 4% *w*/*w* oil phase). Three wall materials were studied: WPI only, OSA only, and a combination of WPI/OSA (50/50 *w*/*w*). The resulting mixtures were then homogenised using a high-speed mixer (L5T, Silverson, East Longmeadow, MA, USA) at 2000 rpm for 5 min to make pre-emulsions. This was followed by homogenization with a pneumatic microfluidizer (HC-2000, Microfluidics Inc., Newton, MA, USA) at 12,000 psi for 3 passes (parameters were selected from initial trials) to obtain three fine emulsions, namely WPI emulsion, OSA emulsion and WO emulsion.

### 2.3. Co-Encapsulation of Carotenoids and Fish Oil

#### 2.3.1. Two-Fluid Nozzle Spray Drying

**Figure 1:**
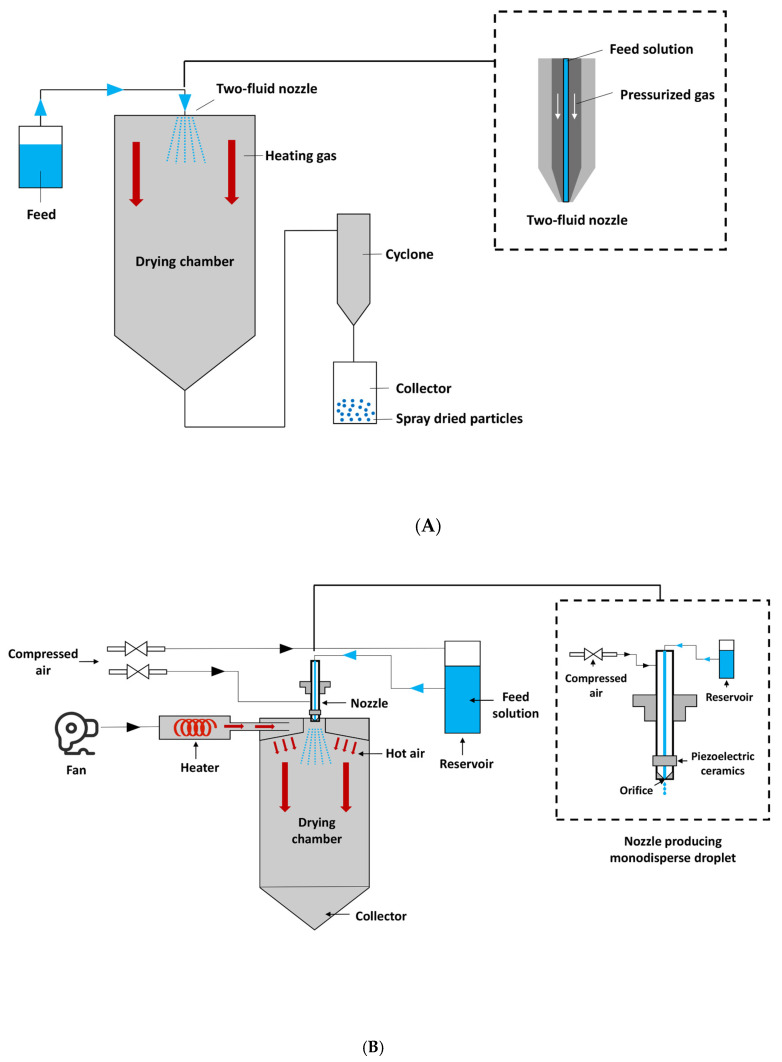
Schematic diagram of (**A**) two-fluid nozzle spray dryer, (**B**) microfluidic-jet spray dryer.

Microencapsulation by SD was conducted using the Buchi Mini Spray Dryer B-191 (Buchi Corporation, Flawil, Switzerland). The schematic diagram of a typical two-fluid nozzle spray dryer was shown in Figure 1A. The inlet temperature was set at 180 °C, the aspirator was at 90% and the pump rate was at 20%. Three microcapsules—SD-W, SD-O, and SD-WO—encapsulated by WPI only, OSA only, and a combination of WPI/OSA (50/50 *w*/*w*), respectively, were obtained. The obtained powder was transferred to a 50 mL centrifuge tube, flushed with nitrogen, and stored in a vacuum desiccator at room temperature until required.

#### 2.3.2. Freeze-Drying

The emulsions were placed in 50 mL centrifuge tubes and were kept at −80 °C for 24 h before being transferred to the freeze dryer (FreezeZone 12 Plus, Labconco, Kansas, MO, USA). During the drying process, the temperature and the vacuum pressure was set at −81 °C and at 0.014 torr, respectively. The dried samples were collected after 72 h of drying, and were further ground into fine powders. Three microcapsules, FD-W, FD-O, and FD-WO using WPI only, OSA only, and a combination of WPI/OSA (50/50 *w*/*w*), respectively, as wall materials, were obtained, and stored following the same procedure as described in Section 2.3.1.

#### 2.3.3. Microfluidic-Jet Spray Drying

The schematic diagram of a typical microfluidic-jet spray dryer is shown in Figure 1B. Feed emulsions were firstly added into the thermostatic heating reservoir of the spray dryer and were kept at 50 °C during the spray drying process. A 75 μm-diameter nozzle atomizer was used for MFJSD in this study. The spray drying conditions was modified according to previous studies [17,18]. The flow pressure was 0.4 psi, inlet temperature was 180 °C, and outlet temperature was controlled in a range of 85–90 °C. The dried powders were collected into 50 mL centrifuge tubes, which were then flushed with nitrogen and stored in desiccator containers at 25 °C until further analyses. Again, three microcapsule samples, MFJSD-W, MFJSD-O, and MFJSD-WO, were produced using WPI only, OSA only, and mixed WPI/OSA (50/50 *w*/*w*), respectively, as wall materials, and they were stored as detailed in Section 2.3.1.

### 2.4. Physiochemical Properties of Microcapsules

#### 2.4.1. Water Activity

Water activity (a_w_) was determined by a water activity analyser (TH-500, Novasina, Switzerland) with sensor temperature set at 25 °C. An appropriate amount of powder sample was placed in the sample pan, and the a_w_ of each sample was obtained when equilibrium was reached.

#### 2.4.2. Density and Flowability

The density of microcapsules was measured according to Rizi et al. [19]. One gram of the microcapsules was transferred to a 10 mL-measuring cylinder. The initial volume was recorded to calculate the bulk density as g/cm^3^. The same cylinder was then tapped until the volume of the microcapsules remained unchanged, and the tapped density (g/cm^3^) was calculated using the second volume. The Carr index was determined by formula (1).

Carr index = [(Bulk density − Tapped density)/Bulk density] × 100%(1)

#### 2.4.3. Microencapsulation Efficiency

Microencapsulation efficiency (ME) of the dried powders was evaluated according to the method by Carneiro et al. [20]. A total of 0.5 g of powder was transferred into a 50 mL centrifuge tube, followed by the addition of 5 mL of hexane. The mixture was gently vortexed for 2 min to extract the surface oil and then filtered with a filter paper (Filter Paper MS2 70 mm, MicroAnalytix). The filtered powder was rinsed three times, each with 5 mL of hexane. The hexane was combined and was further removed from the extracted oil by nitrogen-blow. The encapsulation efficiency was calculated by formula (2).

Microencapsulation efficiency (%) = [(Total oil (g) − Surface oil (g))/Total oil (g)] × 100%(2)

#### 2.4.4. Droplet Size and Particle Size Distribution

The microcapsules were re-dispersed into water, and the droplet size and particle size distribution (PDI) of the resultant dispersions were measured using a Zetasizer Nano ZS (Malvern, Herrenberg, Germany). Each sample was diluted to 0.1% (*w*/*w*) to prevent multiple scattering effects. One millilitre of each diluted sample was added to the cuvette and measured at 25 °C.

### 2.5. Morphological Observation on Microcapsules

The microstructure of the microcapsules was examined using a table top scanning electron microscope (SEM) (Hitachi TM3030Plus, Tokyo, Japan). The samples were placed on the SEM stubs using two-sided adhesive tape and then coated with gold by a desk sputter coater (DSR1, Nanostructured Coatings Co., Tehran, Iran). The coated samples were analysed using the SEM at an accelerating voltage of 15 kV.

### 2.6. Storage Stability of Microcapsules

The storage stability of microcapsules was evaluated via a 4-week storage trial. Samples were placed at a 50 mL centrifuge tubes and stored at 25 or 55 °C. The retention of carotenoids (*β*-carotene, lutein, zeaxanthin), and *ω*-3 LC-PUFAs (EPA, DHA) were determined at week 0 and week 4.

#### 2.6.1. Determination of EPA and DHA

EPA and DHA were determined according to Wei’s method [21]. Briefly, 50 mg of sample was weighed in a 15 mL centrifuge tube, and 50 μL of internal standard methyl heptadecanoate (30 mg/mL) (C_18_H_36_O_2_) was added. Then, 2 mL of the concentrated sulphuric acid/methanol (5%, *v/v*), 300 μL of toluene and 25 μL of BHT/methanol (0.2%, *w*/*w*) were added into the mixture. The mixture was methylated at 92–95 °C for 1.5 h. Afterwards, 2 mL of sodium chloride (0.9%, *w*/*w*) and 2 mL of hexane was added for the extraction of the EPA and DHA methyl esters, which were then determined by gas chromatography (GC), coupled with a flame ionization detector (FID) (Agilent 7890N, Palo Alto, CA, USA) using a HP-FFAP column (30 m × 0.25 mm × 0.25 μm, Agilent J&W GC Columns).

Regarding the GC-FID conditions, nitrogen was used as the carrier gas at a flow rate of 20 mL/min. The oven temperature was set as below: initially at 150 °C; increased from 150 °C to 210 °C at 10 °C /min; held for 7 min; then increased to 230 °C at 20 °C/min and held for 6 min. The injector temperature was 260°C and the split ratio was 30:1. Data were collected using the chemical station software (Agilent, Santa Clara, CA, USA). The concentrations of EPA and DHA were semi-quantified using the internal standard as a reference.

#### 2.6.2. Characterization of Carotenoids

Sample preparation for the determination of *β*-carotene, lutein and zeaxanthin was conducted according to AOAC method (2005.7). Firstly, 50 mg of sample was dissolved in 10 mL of ethanol solution (0.1% *w*/*w* BHT) and 10 mL of KOH solution (10% *w*/*w*) was then added to the sample solution for saponification. The mixture was heated at 70 °C for 10 min. After saponification, 10 mL of a combined petroleum ether and ethanol (50/50%, *v/v*) solution was added to the mixture and vortexed for 3 min. The above solution was further centrifuged at 4500 rpm/min for 5 min. The supernatant was collected and the pellet was re-extracted with 10 mL of the petroleum ether and ethanol solution twice. The extracted oil phases were combined, and the excess solvent was removed under a gentle nitrogen flow. The obtained oil was dissolved in 1 mL of methanol for high performance liquid chromatography (HPLC) analysis.

Quantification of *β*-carotene was performed using an Agilent 1100 series system with a diode array detector-ultraviolent and visible (DAD UV-Vis) detector (Agilent 1260, Agilent Technologies, Santa Clara, CA, USA). A reversed phase C18 column (4.6 × 250 mm, 4 μm, Phenomenex, Torrance, CA, USA) was used. The elution was performed with 98% methanol and 2% acetonitrile from 0 to 25 min. Flow rate was 1.5 mL/min, detection wavelength was 450 nm, and injection volume was 20 μL, and the temperature was 30 °C. HPLC analysis for lutein and zeaxanthin was conducted according to previous studies [22,23], elution was performed with 15% methanol and 85% acetonitrile for 20 min. Flow rate was 0.7 mL/min, detection wavelength was 450 nm, injection volume was 20 μL, and the temperature was 30 °C.

### 2.7. In Vitro Digestion of Microcapsules

Microcapsules were subjected to in vitro digestion experiments simulating oral, gastric and intestinal conditions according to the method from Minekus et al. [24] with slight modification. At the oral phase, 2.5 mL of simulated saliva fluid (SSF) was added into a tube to solubilize a total of 0.5 g of the powder samples. The SSF was composed of SSF electrolyte stock solution (70% *v/v*), α-amylase (1500 U/mL, 10% *v/v*), 0.3 M CaCl_2_ (0.5% *v/v*) and ultrapure water (19.5% *v/v*). The SSF electrolyte solution was constituted by KCl (15.1 mmol/L), KH_2_PO_4_ (3.7 mmol/L), NaHCO_3_ (13.6 mmol/L), MgCl_2_ (0.15 mmol/L) and (NH_4_)_2_CO_3_ (0.06 mmol/L). The oral phase mixture was incubated at 37 °C in a shaking water bath (Ratek, SWB20D, Australia) for 2 min.

At gastric phase, the oral bolus was added by 2.5 mL of simulated gastric fluid (SGF) consisting of SGF electrolyte stock solution (75% *v/v*), pepsin (25,000 U/mL, 16% *v/v*), 0.3 M CaCl_2_ (0.05% *v/v*), ultrapure water (4.55% *v/v*) and 1 mol/L HCl (4.4% *v/v*). The SGF electrolyte solution contained KCl (6.9 mmol/L), KH_2_PO_4_ (0.9 mmol/L), NaHCO_3_ (25 mmol/L), NaCl (47.2 mmol/L), MgCl_2_ (0.1 mmol/L), and (NH_4_)_2_CO_3_ (0.5 mmol/L). The pH of the gastric mixture was adjusted to 3 by 6 mol/L HCl. The mixture was incubated for 2 h at 37 °C. For the intestinal phase, 5 mL of simulated intestinal fluid (SIF) was added to gastric digestion mixture for further intestinal digestion. The SIF was comprised of SIF electrolyte stock solution (55% *v/v*), pancreatic lipase solution (1200 U/mL, 25% *v/v*), bile salt solution (8 mg/mL, 12.5% *v/v*), 0.3 M CaCl_2_ (0.2% *v/v*), ultrapure water (0.75% *v/v*), and its pH was adjusted to 7 with 1 mol/L NaOH (6.55% *v/v*). The SIF electrolyte stock solution was constituted by KCl (6.8 mmol/L), KH_2_PO_4_ (0.8 mmol/L), NaHCO_3_ (85 mmol/L), NaCl (38.4 mmol/L), and MgCl_2_ (0.33 mmol/L). The mixed sample was incubated at 37 °C for 2 h in a shaking incubator.

### 2.8. Digesta Analysis

#### 2.8.1. Droplet Size

The average droplet size of digesta solution from each digestion phase was determined following the same method as described in Section 2.4.4.

#### 2.8.2. Zeta Potential

The zeta potential of digesta solution from each digestion phase was measured using a Zetasizer Nano ZS (Malvern, Herrenberg, Germany). The diluted sample was injected into a capillary cell (Malvern, Herrenberg, Germany), and was measured at 25 °C in the measurement chamber.

#### 2.8.3. Digestion Behaviours

The microstructure of the dried digesta from gastric and intestinal phase was examined by SEM as mentioned in Section 2.5. The digesta after gastric and intestinal phase was also observed by confocal scanning laser microscopy (CSLM) (Olympus FV 1000, Tokyo, Japan) according to Lueamsaisuk’s method [25]. Aliquots from each sample were stained with 5 μL of aqueous Nile Blue (1%, *w*/*w*). The subsample was placed on a microscopy slide with a cover slip. The confocal images were obtained using 10x objective lens. The oil phase was excited with an Argon laser light at 473 nm. The confocal images were acquired by Olympus Fluoview (Viewer 4.2b, Olympus, Japan).

### 2.9. Statistical Analysis

All experiments were conducted in triplicate. Results were expressed as mean ± standard deviation for each sample. One-way ANOVA was used to analyse the data using SPSS Statistics (Version 19, IBM, Armonk, NY, USA). Significance level was determined at *p* < 0.05 (Post hoc test: Duncan’s test).

## 3. Results and Discussion

### 3.1. Microstructure of Microcapsules

**Figure 2:**
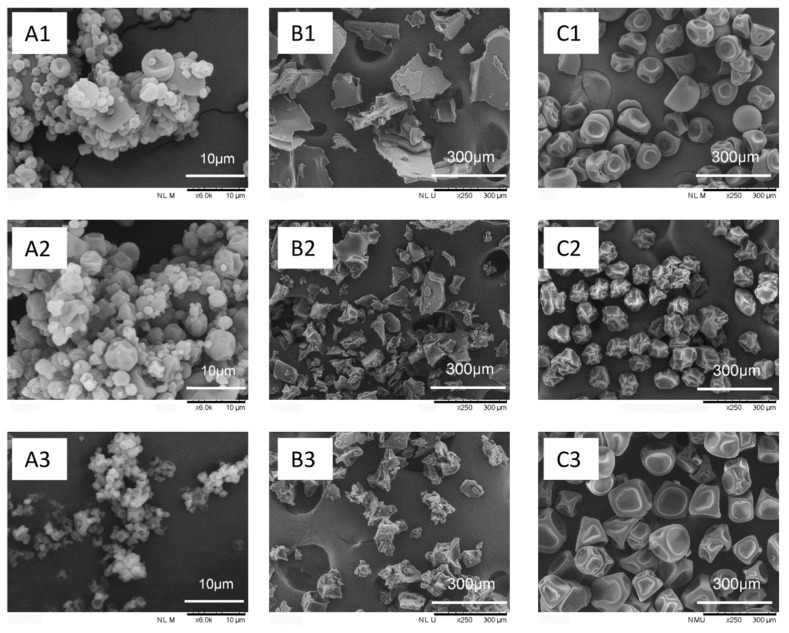
Microstructure of microcapsules. (**A1**) SD-W, (**A2**) SD-O, (**A3**) SD-WO at 6000× magnification; (**B1**) FD-W, (**B2**) FD-O, (**B3**) FD-WO at 250× magnification; (**C1**) MFJSD-W, (**C2**) MFJSD-O, (**C3**) MFJSD-WO at 250× magnification. SD-W, SD-O, and SD-WO: microcapsules produced by two-fluid nozzle spray drying, using WPI only, OSA only, and a combination of WPI/OSA (50/50 *w*/*w*) as wall materials, respectively; FD-W, FD-O, and FD-WO: microcapsules produced by freeze-drying, using WPI only, OSA only, and a combination of WPI/OSA (50/50 *w*/*w*), as wall materials, respectively; MFJSD-W, MFJSD-O, and MFJSD-WO: microcapsules produced by microfluidic-jet spray drying, using WPI only, OSA only, and a combination of WPI/OSA (50/50 *w*/*w*) as wall materials, respectively. WPI: whey protein isolate; OSA: octenylsuccinic anhydride modified starch.

Representative SEM photos of microcapsules produced by three drying methods, FD, SD, and MFJSD, were shown in Figure 2. Overall, the microcapsules obtained by FD (Figure 2; B1, B2, B3) were significantly different from those acquired from SD and MFJSD (Figure 2; A1, A2, A3 and C1, C2, C3, respectively) regarding the particle morphology. The freeze-dried microcapsules showed an irregular and fragmented morphology rather than spherical morphologies seen from the spray-dried powders, and this could be due to the friability of the freeze-dried products.

The MFJSD microcapsules (C1, C2, C3) generally presented identical particle sizes and a good uniformity in morphological characteristics, while the SD microcapsules (A1, A2, A3) showed a wide range of particle size distribution and irregular surface morphologies. Such differences were mostly attributed to the atomization characteristics of the two spraying drying equipment. The atomization of traditional spray dryer is based on a pneumatic nozzle atomizer, which is prone to produce practices with poor homogeneity. However, the MFJSD is equipped with a monodisperse droplet generator, which can produce microcapsules with a good uniformity in both particle size and morphology. It is also noted that the particle size of MFJSD microcapsules (~100 μm) was much larger than that of SD microcapsules (<10 μm) (Figure 2). This result is in agreement with previous findings that the average droplet diameter of MFJSD with an electro-hydrodynamic droplet generator (EHDG) is roughly twice the liquid jet diameter or orifice diameter (75 μm) [26], and the size range of particles produced by two fluid nozzle atomizers is 1–30 μm [27]. Huang et al. [16] also reported that MFJSD microcapsules encapsulated by OSA modified starch showed a particle size ranging between 110 and 180 μm, which was around 10 times larger than those produced by conventional spray dryers [28].

As the droplets were monodispersed by MFJSD, resulting in a minimised aggregation of the powders, the morphology differences among the MFJSD microcapsules (C1, C2, C3) with different wall materials were easily to be observed. For example, the MFJSD-W sample showed round and smooth particle surface with large indents, while the MFJSD-O powder presented irregular surfaces with apparent shrinkage and wrinkles. This could be caused by the different rate of surface crust formation during spray-drying process. It is reported that the OSA emulsion had slower rate of crust formation than the WPI emulsion during the drying process [29,30], and a slower rate of crust formation normally leads to a greater extent of droplet shrinkage before the complete solidification of surface wall and, thus, resulting in more dents and wrinkles on the particle surface [26,27]. Moreover, the morphology of the MFJSD-WO sample was similar to that of the MFJSD-W, both of them presented round and smooth surfaces with large indents. This indicated that WPI had a greater influence on particle morphology than OSA, as the wall material composition of the MFJSD-WO sample was 50% WPI and 50% OSA.

### 3.2. Physicochemical Properties of Microcapsules

#### 3.2.1. Water Activity (a_w_)

In Table 1, the a_w_ range for the microcapsules obtained by each drying technique was 0.241–0.264, 0.234–0.280, and 0.229–0.260 for the SD, FD, and MFJSD samples, respectively. Powder products with a_w_ below 0.6 are generally considered microbial safe; therefore, all microcapsules in this study had high stability against microbial growth. This also suggested that all the three drying techniques employed in this study could achieve good drying performance with a desirable a_w_ value. The microcapsules encapsulated by WPI-only showed higher a_w_ (0.264 for SD-W, 0.280 for FD-W, 0.260 for MFJSD-W) than the OSA microcapsules (0.241 for SD-O, 0.234 for FD-O, 0.229 for MFJSD-O). This may be due to the stronger water binding capacity of OSA modified starch compared to WPI. Yan, et al. [31] reported that high levels of OSA modified starch facilitate the water binding capacity of the oil-in-water emulsion. The lower a_w_ of OSA microcapsules may also result from the high-water binding capacity.

#### 3.2.2. Microencapsulation Efficiency (ME)

As shown in Table 1, the MFJSD microcapsules had the highest ME (94.0–95.1%) followed by the SD samples (84.4–91.7%), and the ME of the FD samples was the lowest (69.9–77.3%) among all. The differences in ME may be related to the morphological characteristics of the microcapsules. The low ME of freeze-dried microcapsules was possibly caused by the high porosity of the particles, which resulted from the sublimation of ice crystals from the inner structure of the frozen samples during freeze-drying.

Compared to the freeze-dried products, spray-dried products showed a higher encapsulation efficiency, which is possibly due to their different drying principles. Specifically, during spray drying, the droplets are ejected into a drying chamber with immediate contact to high temperature air. This process causes instant water evaporation and then solute condensation, which eventually forms a compact and rigid crust for encapsulating the core materials [32]. In addition, the ME of the MFJSD microcapsules was greater than that of the SD microcapsules. This may be related to their particle size. The particle size of the MFJSD microcapsules was much larger than that of the SD microcapsules. Thus, due to a smaller total surface area from a larger particle size, less oil shall present on the surface of the MFJSD microcapsules, resulting in higher ME.

#### 3.2.3. Density and Flowability

The tapped densities of the MFJSD microcapsules (0.37–0.65 g/cm^3^) were higher than those of the SD (0.27–0.46 g/cm^3^) and FD microcapsules (0.32–0.39 g/cm^3^) (Table 1). A low density for powder products generally indicates significant amount of occluded air in the interspace between particles. Literature has also reported that, due to the pneumatic nozzle atomizer, two-fluid nozzle spray dryer is prone to produce particles with high occluded air [32]. Moreover, as mentioned before, the MFJSD microcapsules were over ten-fold larger than the SD microcapsules [33]; therefore, less occluded air would exist in the MFJSD particles, resulting in a higher density of the powders. A low density with more air among particles could also lead to a high risk of oxidation that affects storage stability of powder products. Based on this, the MFJSD microcapsules with a relatively higher powder density were considered more stable during storage.

However, our results on density were not consistent with the findings from Elversson and Millqvist-Fureby [34], who reported a higher density from the spray-dried microcapsules with smaller particle sizes. However, the size difference among their samples produced using different wall materials (approximately 35% to 55%) were much smaller compared to the large size variation (more than 10 times) in our samples. Therefore, the phenomenon in this study could be distinctive, and may require further investigation.

The SD microcapsules showed a higher Carr index (41.0–48.4%) than the MFJSD microcapsules (16.0–30.0%) (Table 1), indicating that the MFJSD microcapsules had a better flowability than the SD microcapsules. The poor flowability of SD microcapsules also can be confirmed by the significantly agglomerated particles, as shown in Figure 1, in the SD microcapsules (A1, A2, A3). In contrast, the MFJSD particles (C1, C2, C3) were well separated and non-agglomerated (Figure 1). Generally, microcapsules with a small particle size would result in a poor flowability because of large surface area per mass unit. The increases on surface area lead to strengthened cohesion and attrition force and result in a flow resistance between particle-particle or particle-container [35]. Furthermore, the irregular droplet trajectories of the SD microcapsules could be another reason for the poor flowability as it can cause complex collisions between particles and/or between particle and the interior dryer surface. Therefore, the MFJSD, equipped with monodisperse droplet generator to produce single stream droplets with similar droplet trajectories and drying paths, could produce microcapsules with enhanced flowability.

Compared to the microcapsules produced by SD and MFJSD, the FD microcapsules had a relatively high Carr index (>34%) probably due to the irregular particle shape and high surface oil present on the surface of the particles. As reported previously in Section 3.2.2, FD microcapsules had a low ME, thereby a high amount of surface oil, which leads to viscous surface and increases attrition force between particles.

#### 3.2.4. Particle Size

**Figure 3:**
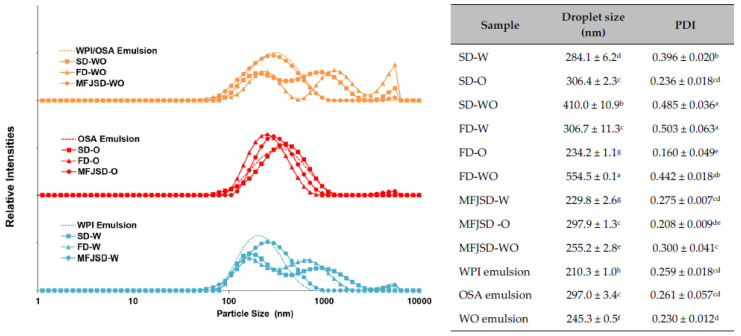
Particle size distribution of the initial and reconstituted emulsions. SD-W, SD-O, and SD-WO: microcapsules produced by two-fluid nozzle spray drying, using WPI only, OSA only, and a combination of WPI/OSA (50/50 *w*/*w*) as wall materials, respectively; FD-W, FD-O, and FD-WO: microcapsules produced by freeze-drying, using WPI only, OSA only, and a combination of WPI/OSA (50/50 *w*/*w*) as wall materials, respectively; MFJSD-W, MFJSD-O, and MFJSD-WO: microcapsules produced by microfluidic-jet spray drying, using WPI only, OSA only, and a combination of WPI/OSA (50/50 *w*/*w*) as wall materials, respectively. WPI: whey protein isolate; OSA: octenylsuccinic anhydride modified starch. WO: a combination of WPI and OSA (50/50 *w*/*w*). PDI: particle size distribution index. Different letters in superscript for each column indicate significant differences at *p* < 0.05.

As shown in Figure 3, the reconstituted emulsions from the MFJSD microcapsules (MFJSD-W, MFJSD-O, MFJSD-WO) presented a unimodal distribution, which was close to its corresponding initial emulsion. While the SD microcapsules (SD-W, SD-WO) and the FD microcapsules (FD-W, FD-WO) showed a bimodal distribution or multimodal distribution after reconstitution. Consistently, compared to the SD and FD microcapsules, the MFJSD microcapsules, after reconstitution, had a droplet size (229.8 nm for MFJSD-W, 297.9 nm for MFJSD-O, and 255.2 nm for MFJSD-WO) much closer to the initial emulsion (210.3 nm for WPI emulsion, 297.0 nm for OSA emulsion, and 245.3 nm for WO emulsion).

In addition, the PDI of the three initial emulsions ranged from 0.230 to 0.261. The MFJSD microcapsules showed a smaller range of PDI in their reconstituted emulsions (0.208–0.300) than the SD microcapsules (0.236–0.485) and the FD microcapsules (0.160–0.503) (Figure 3). These results indicate that the MFJSD microcapsules had a better reconstitution property than the other two kinds of microcapsules. The reason for this phenomenon may be attributed to the good uniformity of MFJSD microcapsules in particle size and morphology. Moreover, it is noted that all OSA microcapsules (SD-O, FD-O, MFJSD-O) showed a unimodal distribution, which was close to the OSA emulsion, suggesting that OSA modified starch is a reliable wall material with good stability and solubility in the reconstitution process regardless of drying methods.

### 3.3. Storage Stability of Microcapsules

**Figure 4:**
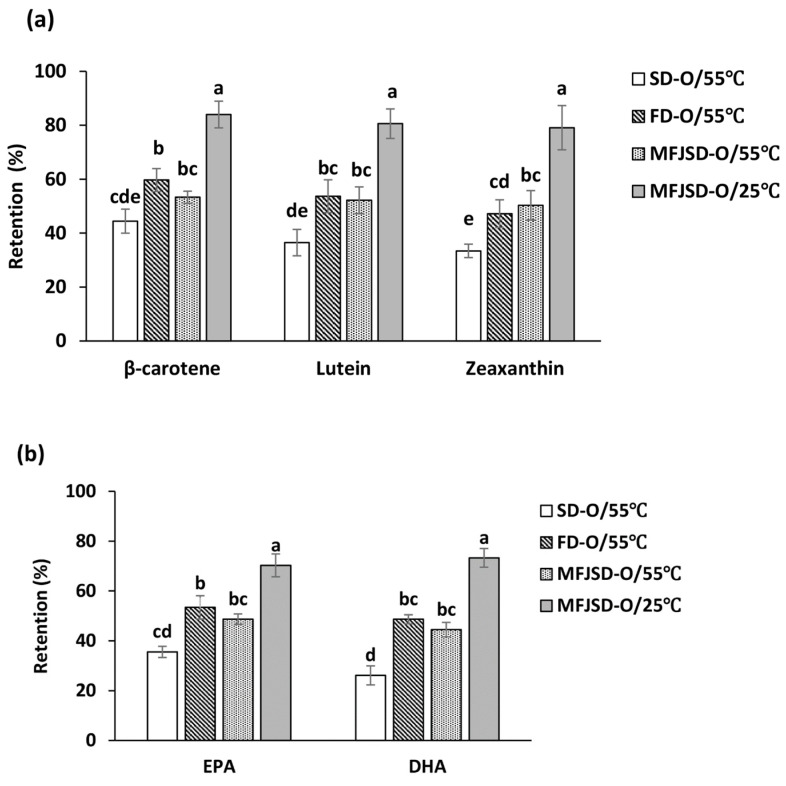
Retentions of (**a**) *β*-carotene, lutein, zeaxanthin and (**b**) EPA, DHA after a 4-week period at 25 °C and 55 °C. SD-O: microcapsules produced by two-fluid nozzle spray drying, using OSA only as wall material; FD-O: microcapsules produced by freeze-drying, using OSA only as wall material; MFJSD-O: microcapsules produced by microfluidic-jet spray drying, using OSA only as wall material. WPI: whey protein isolate; OSA: octenylsuccinic anhydride modified starch. Different lowercase letters indicate significant differences at *p* < 0.05.

After a 4-week storage at 55 °C, the retention of carotenoids and *ω*-3 PUFAs in SD-O microcapsules was lower than that in FD-O and MFJSD-O microcapsules (Figure 4). For example, the retention of *β*-carotene in the SD microcapsules after 4 weeks was 44.5%, which was lower than that in the FD (59.7%) and MFJSD microcapsules (53.3%). The possible reason would be due to a small particle size and large surface area of the SD particles, which increased the possibility of compound degradation/oxidation, as well as the undesired chemical reactions with external substances. Studies have reported that spray-dried powders with a smaller particle size and higher surface areas showed a faster degradation kinetics due to a higher dissolution rate of the embedded active components in larger exposure areas [36,37].

From Figure 4, the MFJSD microcapsules stored at 25 °C had much higher retentions of carotenoids and *ω*-3 PUFAs than those stored at 55 °C, after 4-week storage. Due to the highly unsaturated chemical structure, carotenoids are prone to degrade into volatile and non-volatile compounds when exposed to thermal stress especially in the presence of oxygen. The polyene molecule can be attacked by oxygen either on the *β*-ring or on the chain and subsequently form the initial oxidation products—epoxides [38,39]. Similarly, due to the presence of numerous methylene-interrupted ethylenic double bonds, EPA and DHA are sensitive to heat treatment and could be subjected to a number of chemical transformations, including oxidation, polymerization, cyclization, and double bond migration [40]. In conclusion, MFJSD and FD could provide better protection to bioactive core materials than SD, and both carotenoids and *ω*-3 PUFAs showed a higher stability at a lower storage temperature.

### 3.4. In Vitro Digestion Behaviours of Microcapsules

**Figure 5:**
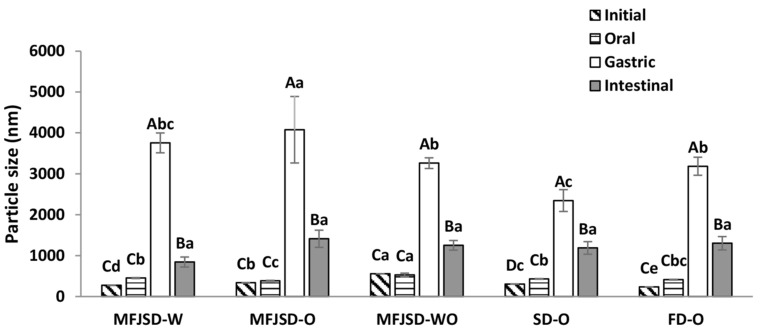
The particle size changes of the microcapsules after in vitro digestion. MFJSD-W, MFJSD-O, and MFJSD-WO: microcapsules produced by microfluidic-jet spray drying, using WPI only, OSA only, and a combination of WPI/OSA (50/50 *w*/*w*) as wall materials, respectively; SD-O: microcapsules produced by two-fluid nozzle spray drying, using OSA only as wall material; FD-O: microcapsules produced by freeze-drying, using OSA only as wall material; WPI: whey protein isolate; OSA: octenylsuccinic anhydride modified starch. The lowercase letters indicate significant difference in different samples with the same digestion phase (*p* < 0.05); the uppercase letters indicate significant difference in same sample (*p* < 0.05).

As can be seen in Figure 5, the particle size of all samples started to increase from oral digestion to gastric digestion, and decreased after intestinal digestion. Particularly, all samples presented a significant increase (*p* < 0.05) of particle size after gastric digestion. Further, after intestinal digestion, the particle size of all samples, although still larger than the initial emulsions, showed a significant decrease (*p* < 0.05).

The slight changes on the particle size after the digestion in oral phase could be due to the altered ionic strength and pH value in samples [41]. Furthermore, the markedly increase of the particle size diameter after a subsequent digestion in the stomach phase resulted from the changes in pH and ionic strength, as well as the effect of the hydrolysed products from the adsorbed proteins and polysaccharides [42,43]. The low pH in the stomach phase caused a decrease of the absolute zeta potential on emulsion droplets, which led to the aggregation of droplets by weakening the electrostatic repulsion force.

Another reason for droplet size increase was due to the presence of pepsin and *α*-amylase in the digestion system. After WPI and OSA modified starch underwent enzymolysis, their chemical structures were altered and their emulsifying abilities were weakened, which led to the occurrence of agglomeration and, thereby, the increase of droplet size. After digestion in the intestinal phase, the particle size of all samples showed a decrease compared to that in the stomach phase. This may be due to the increase of pH, and effect from the lipid hydrolysis by pancreatin and bile salts in the system. The increase of pH would result in a larger absolute zeta potential on droplets and, thereby, a stronger electrostatic repulsion force to keep droplets from aggregation or coalescence, finally leading to the decrease of droplet size [44].

**Figure 6:**
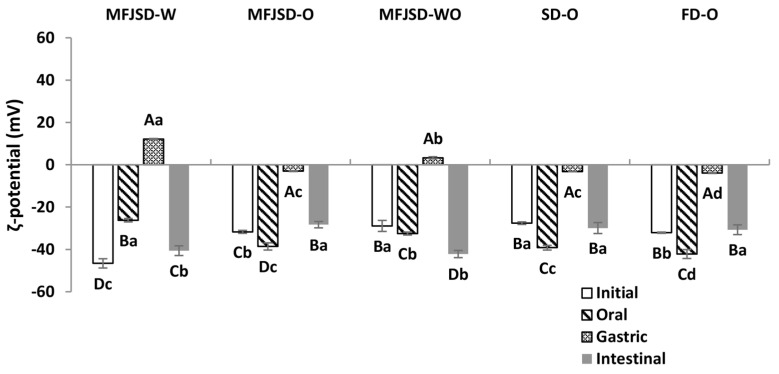
Zeta potential of microcapsules after in vitro digestion. MFJSD-W, MFJSD-O, and MFJSD-WO: microcapsules produced by microfluidic-jet spray drying, using WPI only, OSA only, and a combination of WPI/OSA (50/50 *w*/*w*) as wall materials, respectively; SD-O: microcapsules produced by two-fluid nozzle spray drying, using OSA only as wall material; FD-O: microcapsules produced by freeze-drying, using OSA only as wall material; WPI: whey protein isolate; OSA: octenylsuccinic anhydride modified starch. The lowercase letters indicate significant difference in different samples with the same digestion phase (*p* < 0.05); the uppercase letters indicate significant difference in same sample (*p* < 0.05).

After passing through the intestinal phase, the MFJSD-O sample showed a larger droplet size diameter (1410.5 nm) than the MFJSD-W (843.0 nm) (*p* > 0.05). The difference in the droplet size between these two MFJSD microcapsules after the intestinal digestion was mainly due to the different polymer properties of the two wall materials, WPI and OSA. There was a difference in zeta potential of digested emulsions between MFJSD-O and MFJSD-W. In Figure 6, the absolute zeta potential of the MFJSD-O sample after intestinal digestion was 28.3 mV, which was lower than that of the MFJSD-W (40.6 mV). As reported by previous studies, the presence of electrical charges on droplet surface may contribute to a strong electrical repulsion to prevent droplets against aggregation and flocculation [45,46].

Moreover, there was no significant difference (*p* > 0.05) in droplet size of emulsions after the intestinal digestion among MFJSD-O, SD-O, and FD-O (Figure 5). This phenomenon may be explained by the fact that the three samples were dried from the same original emulsion, which is very likely to be digested at a comparable degree reflecting a similar droplet size of the final digested emulsions.

**Figure 7:**
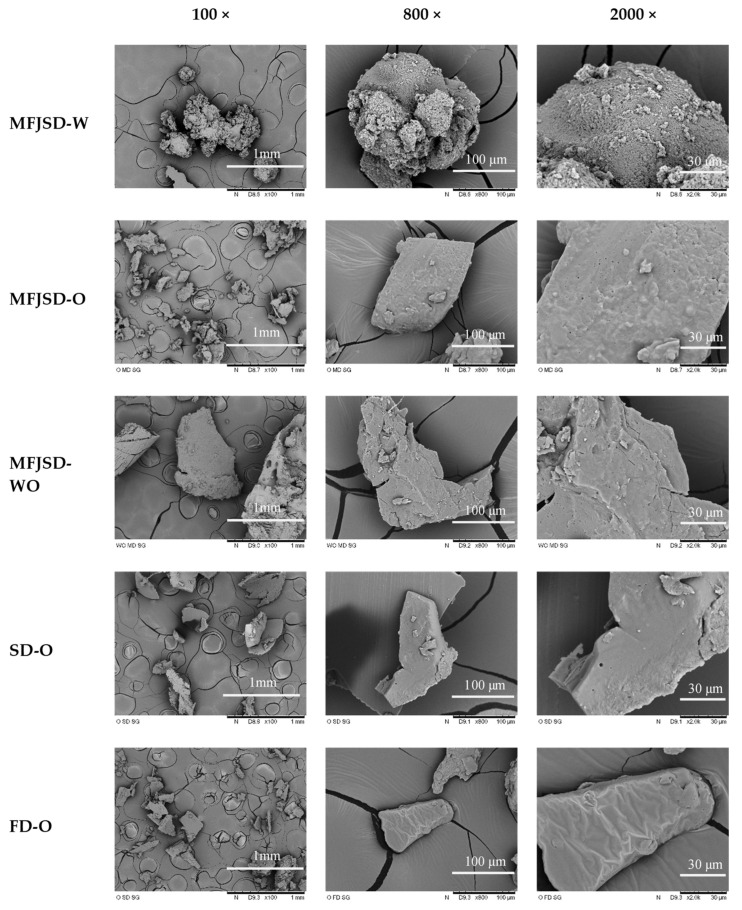
Microstructure of microcapsules after simulated gastric digestion (magnification: 100×, 800×, 2000×). MFJSD-W, MFJSD-O, and MFJSD-WO: microcapsules produced by microfluidic-jet spray drying, using WPI only, OSA only, and a combination of WPI/OSA (50/50 *w*/*w*) as wall materials, respectively; SD-O: microcapsules produced by two-fluid nozzle spray drying, using OSA only as wall material; FD-O: microcapsules produced by freeze-drying, using OSA only as wall material; WPI: whey protein isolate; OSA: octenylsuccinic anhydride modified starch.

It can be seen from Figure 7 that after the gastric digestion, the MFJSD-W sample retained its spherical shape under the effect of protease (pepsin), but the surface of the MFJSD-W sample decomposed from a relatively smooth surface into a rough surface. There were also some digested particles in randomised sizes attached on its spherical surface. For the samples containing OSA modified starch (MFJSD-O, MFJSD-WO, SD-O, FD-O), they all lost their spherical shape (Figure 7) due to the effect of amylase, and after gastric digestion, the shape were further reorganised and became irregular.

**Figure 8:**
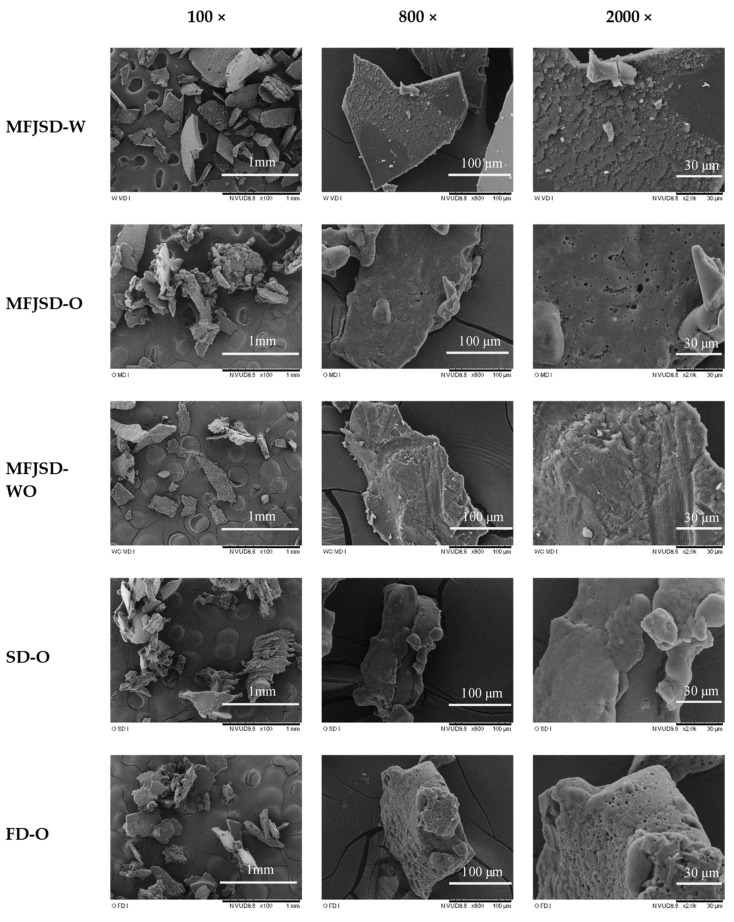
Microstructure of microcapsules after simulated intestinal digestion (magnification: 100×, 800×, 2000×). MFJSD-W, MFJSD-O, and MFJSD-WO: microcapsules produced by microfluidic-jet spray drying, using WPI only, OSA only, and a combination of WPI/OSA (50/50 *w*/*w*) as wall materials, respectively; SD-O: microcapsules produced by two-fluid nozzle spray drying, using OSA only as wall material; FD-O: microcapsules produced by freeze-drying, using OSA only as wall material; WPI: whey protein isolate; OSA: octenylsuccinic anhydride modified starch.

After further digestion in the simulated intestines (Figure 8), the MFJSD-W sample finally lost its original spherical shape because of the effect of protease contained in the pancreatin, which had further digested and decomposed whey proteins in the MFJSD-W sample. On the other hand, there were more holes appearing on the surface of the OSA-containing particles after the intestinal digestion. These pores may be due to the digestion effect of amylase in the added pancreatin, contributing to the release of oil droplets to the surface of the particles.

Moreover, the MFJSD sample, containing WPI (MFJSD-W), showed a sharper and crumbly morphology compared to the samples containing OSA modified starch (MFJSD-O, MFJSD-WO, SD-O, FD-O) after intestinal digestion (Figure 8). This may be because the dried particles containing whey proteins tend to have a hard crust surface with a low elasticity [29]. To the contrary, the particle surface of the OSA-containing samples presented as more rounded and softer, which may be due to the high elasticity of OSA modified [47].

**Figure 9:**
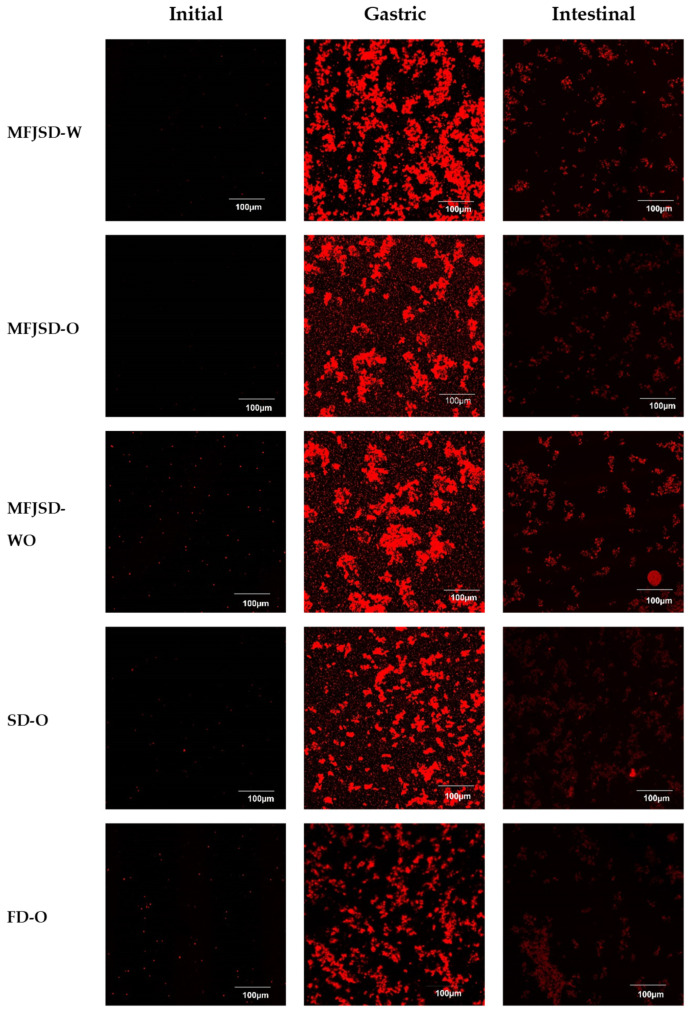
CLSM images showing the digestion behaviours of oil droplets in microcapsules after in vitro gastric and intestinal digestion. MFJSD-W, MFJSD-O, and MFJSD-WO: microcapsules produced by microfluidic-jet spray drying, using WPI only, OSA only, and a combination of WPI/OSA (50/50 *w*/*w*) as wall materials, respectively; SD-O: microcapsules produced by two-fluid nozzle spray drying, using OSA only as wall material; FD-O: microcapsules produced by freeze-drying, using OSA only as wall material; WPI: whey protein isolate; OSA: octenylsuccinic anhydride modified starch.

In order to have a clearer view on the digestion behaviour of the microcapsules at different stages, samples were stained to show the undigested oil droplets. As shown in Figure 9, all samples showed a similar behaviour from the initial stage to the gastric stage and intestinal stage. More specifically, all samples had very few oil droplets to be stained by Nile blue (shown as red colour) at the initial stage indicating the oil droplets were well encapsulated by the emulsifying materials at this stage. The dye cannot touch the oil droplets because the macromolecules of the wall materials, WPI and OSA, adhered to the surface of the oil droplets due to their lipophilicity.

In the simulated stomach stage, under the effect of α-amylase and pepsin, the WPI and OSA in the emulsion were gradually decomposed, and simultaneously the enclosed oil droplets were gradually released to react with the dye to develop colour. Therefore, in the simulated stomach stage, we can observe a large number of oil droplets were stained and many of them aggregated (Figure 9). In the simulated intestine stage, the oil droplets released from the simulated stomach stage were gradually broken down by the pancreatic lipase. The decomposed fatty acids and bile salts were combined to form micelles, which were used for subsequent absorption by intestinal wall cells.

## 4. Conclusions

In this study, three microencapsulation techniques, namely microfluidic-jet spray drying (MFJSD), two-fluid nozzle spray drying (SD), and freeze-drying (FD), were employed to produce microcapsules for a comparison on the morphology, physiochemical properties, storage stabilities, and digestion behaviours of the powders. Three different types of wall materials, WPI only, OSA only, and WPI/OSA 50%/50%, were also applied to each drying method. As a result, the MFJSD microcapsules showed the higher microencapsulation efficiency, higher flowability, and better reconstitution properties than SD and FD microcapsules, mainly due to the good uniformity in the morphology and particle size. After 4-week storage at 55 °C, MFJSD and FD microcapsules had higher retentions on carotenoids and *ω*-3 PUFAs than SD microcapsules because of the smaller particle size of the spray-dried microcapsules. Moreover, through in vitro digestion trials, it was found that the differences in digestion behaviours of microcapsules were mainly dependent on their wall materials rather than microencapsulation techniques. This study has systematically investigated the co-encapsulation of carotenoids and fish oil by the MFJSD with comparison to the other two commonly used microencapsulation techniques. The results showed the feasibility of the application of the MFJSD for the encapsulation of lipophilic bioactives. It also provided important information on how different atomization mechanisms and drying techniques affect the properties of the final microcapsules.

## Figures and Tables

**Table 1:** Physicochemical properties of microcapsules.

Samples	Water Activity	ME (%)	Tapped Density (g/cm^3^)	Carr Index (%)
SD-W	0.264 ± 0.002 ^bc^	84.4 ± 0.1 ^b^	0.27 ± 0.01 ^d^	44.6 ± 3.0 ^a^
SD-O	0.241 ± 0.010 ^bcd^	91.7 ± 0.2 ^a^	0.46 ± 0.02 ^b^	41.0 ± 3.7 ^a^
SD-WO	0.260 ± 0.000 ^abc^	85.4 ± 1.8 ^b^	0.32 ± 0.00 ^cd^	48.4 ± 1.4 ^a^
FD-W	0.280 ± 0.008 ^a^	69.9 ± 4.9 ^d^	0.32 ± 0.01 ^cd^	34.1 ± 1.4 ^b^
FD-O	0.234 ± 0.028 ^cd^	77.3 ± 3.8 ^c^	0.39 ± 0.00 ^c^	34.0 ± 2.5 ^b^
FD-WO	0.236 ± 0.010 ^cd^	74.3 ± 0.2 ^cd^	0.33 ± 0.02 ^cd^	43.1 ± 6.5 ^a^
MFJSD-W	0.260 ± 0.001 ^abc^	94.0 ± 1.5 ^a^	0.46 ± 0.01 ^b^	30.0 ± 1.0 ^b^
MFJSD -O	0.229 ± 0.006 ^d^	94.1 ± 0.6 ^a^	0.65 ± 0.01 ^a^	19.2 ± 1.0 ^c^
MFJSD-WO	0.231 ± 0.004 ^d^	95.1 ± 0.7 ^a^	0.37 ± 0.03 ^c^	16.0 ± 2.7 ^c^

SD-W, SD-O, and SD-WO: microcapsules produced by two-fluid nozzle spray drying, using WPI only, OSA only, and a combination of WPI/OSA (50/50 *w*/*w*) as wall materials, respectively; FD-W, FD-O, and FD-WO: microcapsules produced by freeze-drying, using WPI only, OSA only, and a combination of WPI/OSA (50/50 *w*/*w*) as wall materials, respectively; MFJSD-W, MFJSD-O, and MFJSD-WO: microcapsules produced by microfluidic-jet spray drying, using WPI only, OSA only, and a combination of WPI/OSA (50/50 *w*/*w*) as wall materials, respectively. WPI: whey protein isolate; OSA: octenylsuccinic anhydride modified starch. ME: microencapsulation efficiency; Different letters in superscript for each column indicate significant differences at *p* < 0.05.

## Data Availability

The data presented in this study are available upon request from the first author.
